# Supplementary material for: Is the A-1 Pigment in Photosystem I Part of P700? A (P700+–P700) FTIR Difference Spectroscopy Study of A-1 Mutants
Source: Int J Mol Sci. 2024 Apr 29;25(9):4839. doi: 10.3390/ijms25094839 (PMC11084411; doi:10.3390/ijms25094839)
Supplement: Supplementary file 1 [file ijms-25-04839-s001.zip › ijms-2973193-supplementary.pdf]

Supporting Information

The A_{-1} Pigment Photosystem I Contributes to ($P700^+$ – P700) FTIR Difference Spectra: Cyanobacterial PSI.

Julia S. Kirpich,[1] Lujun Luo,[2] Michael R. Nelson,[1] Neva Agarwala,[1] Wu Xu[2] and Gary Hastings[1]

[1]Department of Physics and Astronomy, Georgia State University, Atlanta, GA 30303, USA

[2]Department of Chemistry, University of Louisiana at Lafayette, Lafayette, LA 70504, USA

*Corresponding author, Gary Hastings, Email: ghastings@gsu.edu

[3]Current Address: Department of Chemistry, Georgia State University, Atlanta, GA 30303, USA

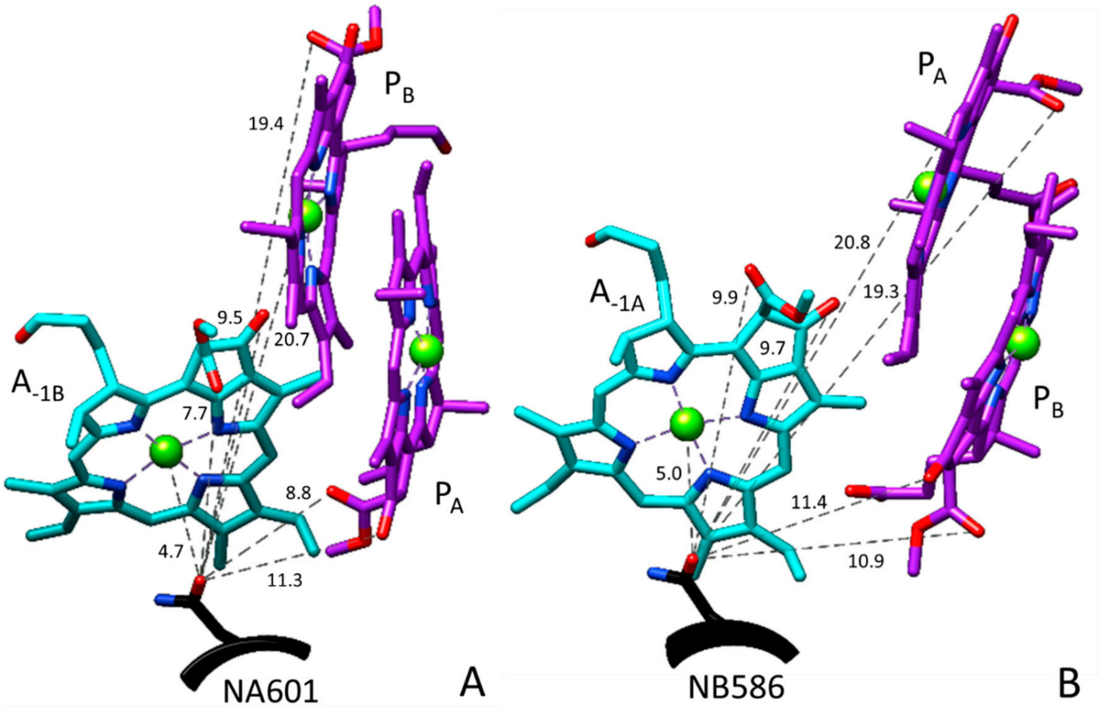

Figure S1. Structural organization of the (A) A_{-1B}, and (B) A_{-1A} pigments. The P_A and P_B pigments are also shown. P_A and P_B are in purple and $A_{-1A/B}$ are in cyan. Structure obtained using the cryo-electron microscopy structure of PSI from *C. reinhardtii* at 2.90 Å resolution (PDB file 6JO5) [1]. Asn at A601 and B586 are also shown (*backbone in black*). Distances from the Asn carbonyl oxygen atoms are shown (*dashed lines*). Distances are in Å and are also listed in Table S1.

Table S1. Distances (in Å) between the C=O oxygen atom of Asn and various atoms of molecular groups of the P_A and P_B pigments in PSI from *C. reinhartdtii* (6JO5).

	**Asn carbonyl oxygen atom (NB586) SEE FIGURE S1B**	**Asn carbonyl oxygen atom (NA601) SEE FIGURE S1A**
H_2O oxygen atom	-	-
Mg atom	5.0	4.7
13^1-keto carbonyl oxygen atom of P_A	20.8	11.3
13^3-ester carbonyl oxygen atom of P_A	19.3	8.8
13^1-keto carbonyl oxygen atom of P_B	11.4	20.7
13^3-ester carbonyl oxygen atom of P_B	10.9	19.4
13^1-keto carbonyl oxygen atom of A_{-1A}	**9.7**	15.2 (not in Fig.2)
13^3-ester carbonyl oxygen atom of A_{-1A}	**9.9**	18.4 (not in Fig.2)
13^1-keto carbonyl oxygen atom of A_{-1B}	15.2 (not in Fig.2)	**9.5**
13^3-ester carbonyl oxygen atom of A_{-1B}	18.3 (not in Fig.2)	**7.7**

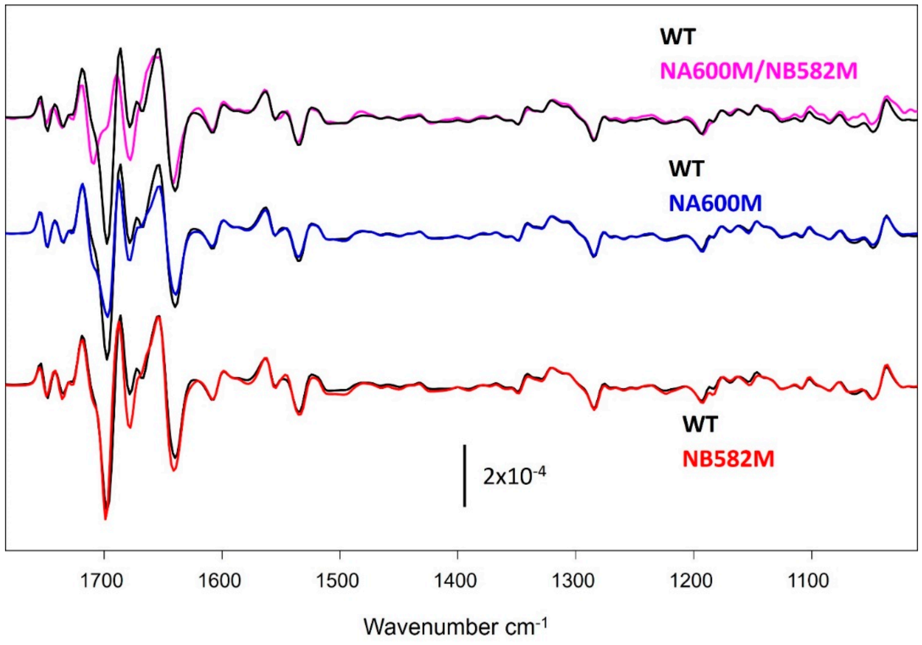

Figure S2. RT (P700$^+$- P700) FTIR DS for WT and mutant PSI samples in the 1800-1050 cm^{-1} region. The same WT FTIR DS (black) is shown overlaid with each mutant FTIR DS. FTIR DS are the average of three measurements. The WT and mutant spectra were scaled so the 1748(-)/1754(+) cm^{-1} difference bands were similar. The data in the figure indicate that such a scaling minimizes the differences between the DS over the entire spectral region. Absorbance difference of 2x10^{-4} (in OD units) is indicated by the black bar.

REFERENCES

1. Suga, M., et al., *Structure of the green algal photosystem I supercomplex with a decameric light-harvesting complex I.* Nature Plants, 2019. **5**(6): p. 626-636.
